# Flavour, emulsifiers and colour are the most frequent markers to detect food ultra-processing in a UK food market analysis

**DOI:** 10.1017/S1368980023002185

**Published:** 2023-12

**Authors:** Nathalie Judith Neumann, Gerrit Eichner, Mathias Fasshauer

**Affiliations:** 1 Institute of Nutritional Science, Justus-Liebig University of Giessen, Goethestr. 55, Giessen, Hessen 35390, Germany; 2 Mathematical Institute, Justus-Liebig University of Giessen, Giessen, Germany; 3 Center for Sustainable Food Systems, Justus-Liebig University of Giessen, Giessen, Germany

**Keywords:** Food additives, Markers of ultra-processing, NOVA classification, Ultra-processed food

## Abstract

**Objective:**

To elucidate which markers of ultra-processing (MUP) and their combinations are best suited to detect ultra-processed food (UPF).

**Design:**

The study was based on the 206 food and 32 beverage items of the Oxford WebQ which encompass all major foods consumed in the UK. For each Oxford WebQ question, ingredient lists of up to ten matching different commercial products (*n* 2146) were researched online using data from the two market leaders of groceries in the UK sorted by relevance (Tesco) and by top sellers (Sainsbury’s), respectively. According to the NOVA classification, sixty-five MUP were defined, and if the ingredient list of a food product was positive for at least one MUP, it was regarded as UPF. The percentage of UPF items containing specific MUP was calculated. In addition, all combinations of two to six different MUP were assessed concerning the percentage of identified UPF items.

**Setting:**

Cross-sectional analysis.

**Participants:**

None.

**Results:**

A total of 990 products contained at least one MUP and were, therefore, regarded as UPF. The most frequent MUP were flavour (578 items, 58·4 % of all UPF), emulsifiers (353 items, 35·7 % of all UPF) and colour (262 items, 26·5 % of all UPF). Combined, these three MUP detected 79·2 % of all UPF products. Detection rate increased to 88·4 % of all UPF if ingredient lists were analysed concerning three additional MUP, that is, fibre, dextrose and firming agent.

**Conclusions:**

Almost 90 % of all UPF items can be detected by six MUP.

Over the decades, the rising prevalence of obesity has become a major public health threat that is increasingly evident around the world^(1)^. Obesity has a significant impact on the global incidence of CVD, type 2 diabetes mellitus, cancer, osteoarthritis, work disability and sleep apnoea^(1)^.

A strong positive link exists between the consumption of ultra-processed food (UPF) and the risk of overweight and obesity^(2)^. UPF is defined as ready-to-consume or heat-up food items high in fat, salt and sugar, as well as low in dietary fibre, protein and micronutrients, usually packaged attractively and marketed intensively^(3)^. Ultra-processing is used to create products that are convenient, hyper-palatable, and highly profitable and can replace other food groups^(3)^. There is a substantial expansion in the types and quantities of UPF sold worldwide, representing a transition towards a more highly processed global diet^(4)^. Limiting highly processed food or UPF has been recommended in several nutrition guidelines, including Brazil^(5)^, Canada^(6)^, Ecuador^(7)^, France^(8)^, Israel^(9)^, Japan^(10)^, New Zealand^(11)^ and Peru^(12)^.

Industrial food processing is assessed by the NOVA classification, a food-rating scheme based on the extent and purpose of processing and, thus, distance from nature, which classifies food and food products into four groups^(3,13)^. Processing according to the NOVA classification includes physical, biological and chemical methods during the manufacturing process^(3)^. Unprocessed and minimally processed food together form NOVA group 1^(13)^. NOVA group 2 contains processed culinary ingredients like oil, sugar and salt which are obtained from group 1 or directly from nature^(13)^. Processed industrial products made by adding those culinary ingredients belong to NOVA group 3^(13)^. UPF is defined as NOVA group 4, and several cosmetic additives, as well as non-culinary ingredients, are exclusively found in this group^(13)^. Cosmetic additives, for example, flavours, colouring agents and sweeteners, make the final products more palatable or appealing^(13)^. Non-culinary ingredients are food substances never or rarely used in the kitchen, for example, varieties of sugars such as dextrose or fructose, modified oils and protein sources such as casein or soya protein isolates^(13)^. The use of these additives and ingredients can mask undesirable sensory characteristics and improve sensory properties^(13)^. A food product is defined as NOVA group 4 if its ingredient list contains at least one cosmetic additive or non-culinary ingredient^(13)^. Even in the absence of cosmetic additives or non-culinary ingredients, a food item is also regarded as UPF if some drastic processes are applied directly to the food, for example, extrusion, hydrogenation, hydrolysation, moulding, pre-frying or puffing^(3,13,14)^.

Focusing on cosmetic additives and non-culinary ingredients has been suggested as one approach to simplify the assessment of UPF^(13)^. Davidou and co-workers were the first to define a specific and exhaustive list of markers of ultra-processing (MUP) for the NOVA-based Siga classification^(14)^. However, more than 100 different MUP have been described^(3,13,14)^ which makes the detection of UPF difficult. Therefore, the present study elucidates which MUP and combinations of them are best suited to detect UPF in a UK food market analysis.

## Methods

### Search strategy to select food products

All assessments concerning MUP were based on the Oxford WebQ. The Oxford WebQ is an online dietary questionnaire assessing food and beverage intake from the previous day^(15)^. Similar to a 24-h dietary recall by an interviewer, the Oxford WebQ provides quantitative information on all foods and beverages consumed^(15)^. A total of 206 food and 32 beverage items are assessed in the Oxford WebQ^(16)^. These food and beverage items encompass all major foods consumed in the UK and, therefore, the Oxford WebQ is best suited to study UK populations^(15)^. Food items are evaluated independent from specific brands, for example, intake of ‘chocolate biscuits (e.g. choc chip cookies, chocolate digestive biscuits)’ is assessed but not consumption of specific chocolate biscuits brands.

For each Oxford WebQ question, ingredient lists of up to ten matching commercial products from the two market leaders of groceries in the UK, that is, Tesco and Sainsbury’s^(17)^, were analysed. Online research within the subcategories best matching the Oxford WebQ questions was primarily done at Tesco (https://www.tesco.com/groceries/en-GB) and in case of fewer than ten items found there also at Sainsbury’s (https://www.sainsburys.co.uk/shop/gb/groceries). Food products within the chosen categories were sorted by relevance (Tesco) or by top sellers (Sainsbury’s). The first ten products matching the Oxford WebQ questions were chosen. If different flavours of the same brand, for example, chocolate and strawberry ice cream of the same brand, were listed within these top ten, they were included in the analysis. In case of fewer than ten matching products found at Tesco and Sainsbury’s combined, all available products were included in the analysis.

The Oxford WebQ has been developed and validated for adult participants only^(18–20)^. Accordingly, the Oxford WebQ has been exclusively used in large studies with adult participants^(15)^. Therefore, food specifically targeting children, for example, having the words ‘kids’ or ‘children’ on the packaging, was excluded from the present analysis.

### Ingredient lists of food products

In total, 2146 different products were analysed in the present study concerning the 238 Oxford WebQ items. If no ingredient lists were given, a further internet search was performed on www.amazon.co.uk and other product information sites. For 310 food products, no ingredient lists could be found. These include unprocessed meat, fish, fruit, vegetables, eggs, oils, nuts, and seeds, as well as water and alcoholic beverages such as wine and spirits. European law does not require mandatory provision of ingredient lists for these types of food^(21)^. Items without ingredient lists were excluded from further analysis. The ingredient lists of the remaining 1836 food products were recorded.

### MUPs

According to two recent publications by Monteiro and co-workers^(3,13)^, sixty-five MUP were defined based on nine categories, that is, flavours, flavour enhancers, colouring agents, sweeteners, processing aids, varieties of sugar, modified oils, protein sources, and fibres, and their individual compounds (see online Supplemental Table 1). All MUP were optimised for maximal detection of individual compounds, for example, the MUP ‘glutam*’ identifies individual compounds such as glutamic acid, monosodium glutamate and magnesium diglutamate (see online Supplemental Table 1). Ingredient lists of the 1836 food products were analysed concerning the sixty-five MUP. If a food product was positive for at least one MUP, it was regarded as UPF. If an additive was not a MUP in all circumstances, for example, maltodextrin is a MUP if used as a bulking agent but not if used as a stabiliser (i.e. in this context a marker for NOVA group 3^(3)^), it was regarded as a MUP nevertheless. If a MUP could be classified into different functional classes, for example, maltodextrin in the categories processing aids and varieties of sugar, the decision about the most appropriate category was reached by the consensus of all authors.

### Statistical analysis

All data analyses and graphical representations were performed using R version 4.0.5. The sixty-five MUP were extracted from the ingredient lists and sorted by frequency. The percentage of UPF items containing specific MUP was calculated. In addition, all combinations of two to six different MUP were assessed, and the percentage of UPF items identified by these combinations was calculated. The respective combinations with the highest UPF detection rate were graphically represented using Venn diagrams.

## Results

### Frequency of single MUP in UPF

Of the 1836 food products with ingredient lists, 990 (53·9 %) were positive for at least one MUP and, therefore, defined as UPF. Within these 990 UPF products, forty-three MUP were detected at least once, whereas twenty-two MUP were not found in the ingredient lists (Table 1). The most frequent MUP with a frequency >10 % of all UPF were flavour (578 products, 58·4 % of all UPF), emulsif* (353 products, 35·7 % of all UPF), colour (262 products, 26·5 % of all UPF), dextrose (163 products, 16·5 % of all UPF), whey (145 products, 14·6 % of all UPF) and gluten (100 products, 10·1 % of all UPF) (Table 1). From the MUP identified in at least one product, a total of twenty-four and thirteen MUP were present in 1 % to 10 % and in less than 1 % of UPF, respectively (Table 1).

**Table 1:** MUP sorted by frequency^†^

Ranking	MUP	Frequency	% UPF^‡^
1	Flavour	578	58·4
2	Emulsif^*^	353	35·7
3	Colour	262	26·5
4	Dextrose	163	16·5
5	Whey	145	14·6
6	Gluten	100	10·1
7	Fibre	93	9·4
8	Barley malt extract	79	8·0
9	Sweetener	76	7·7
10	Maltodextrin	74	7·5
11	Fructose	72	7·3
12	Gelling	65	6·6
13	Thickener	64	6·5
14	Humectant	63	6·4
15	Invert^*^	62	6·3
16	Lactose	56	5·7
17	Firming	44	4·4
18	Caking	28	2·8
19	Sucralose	28	2·8
20	Acesulfame	27	2·7
21	Glazing	26	2·6
22	Flavour enhancer	25	2·5
23	Glutam^*^	20	2·0
24	Steviol	20	2·0
25	Aspartame	17	1·7
26	Ribonucleotide^*^	17	1·7
27	Isomalt	16	1·6
28	Sorbitol	14	1·4
29	Bulking	10	1·0
30	Hydrolysed	10	1·0
31	Maltitol	8	0·8
32	Saccharin	8	0·8
33	Erythritol	7	0·7
34	Hydrogenated	7	0·7
35	Isolate^*^	5	0·5
36	Inosin^*^	4	0·4
37	Guanyl^*^	3	0·3
38	E95^*^	2	0·2
39	Sequestrant	2	0·2
40	E62^*^	1	0·1
41	E63^*^	1	0·1
42	Foaming	1	0·1
43	Xylitol	1	0·1

* All variations after the word are possible (e.g. glutamic and glutamate are possible for glutam*).

† The following twenty-two MUP were not found in any of the ingredient lists: advantame, carbonating, casein, cyclam*, dye, E420, E421, E640, E650, E96*, glycine, interesterified, lactitol, maltol, mannitol, mechanically separated meat, msg, neohesperidine, neotame, polyglycitol, thaumatin and zinc acetate.

‡ % UPF indicates which percentage of UPF items (*n* 975) were positive for the respective MUP.

### Combinations of MUP

**Fig. 1:**
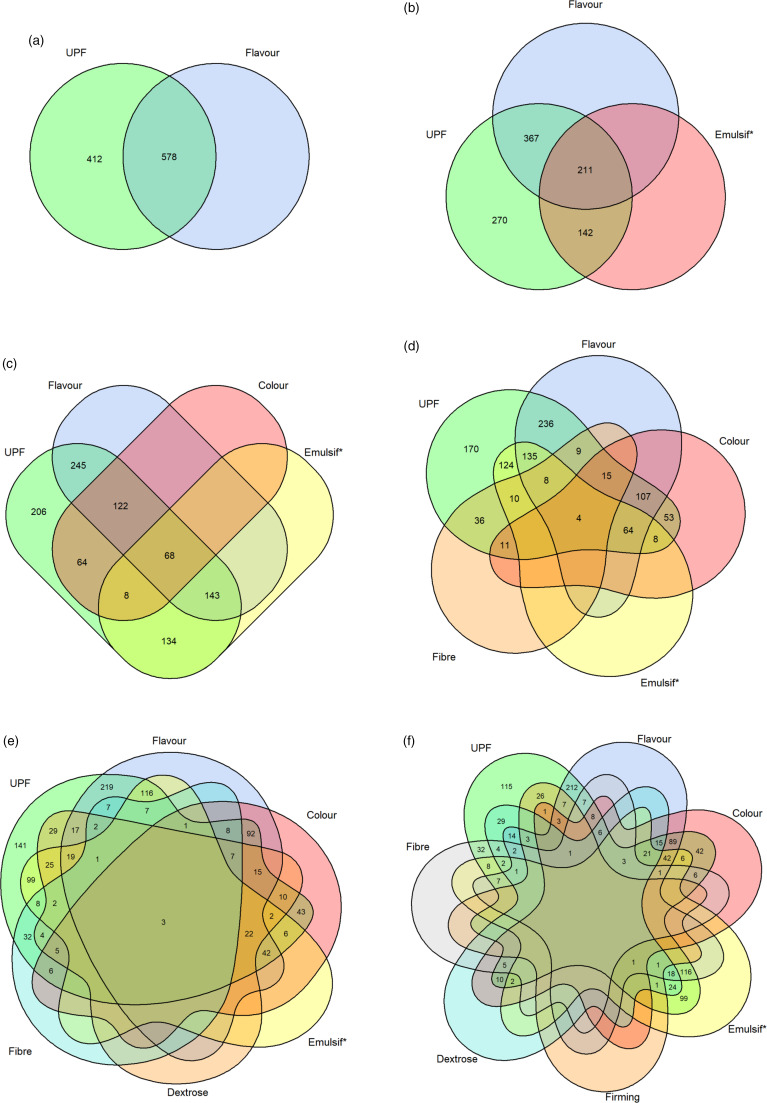
Venn diagrams depicting combinations of (a) one, (b) two, (c) three, (d) four, (e) five and (f) six MUP which detected the maximum number of UPF products (total *n* 990)

Flavour was the MUP detecting the highest proportion of UPF products (578 of 990; 58·4 %; Table 1 and Fig. 1(a)). Next, it was assessed how many UPF products can be detected maximally by two to six combinations of MUP. The most successful combination of two was flavour and emulsif*. Thus, flavour and emulsif* alone detected 367 and 142 UPF items, respectively, and the combination of both terms detected an additional 211 UPF items, resulting in a total of 720 out of 990 UPF products (72·7 %; Fig. 1(b)). The most successful combination of three was flavour, emulsif* and colour detecting 784 UPF items (i.e. 245 flavour alone, 134 emulsif* alone, 64 colour alone, 143 combination of flavour and emulsif*, 122 combination of flavour and colour, 8 combination of emulsif* and colour, 68 combination of flavour, emulsif*, and colour; 79·2 %; Fig. 1(c)). With the most successful combination of four, that is, flavour, emulsif*, colour and fibre, 820 UPF products were detected (82·8 %, Fig. 1(d)). The most successful combination of five consisting of flavour, emulsif*, colour, fibre and dextrose detected 849 UPF items (85·8 %, Fig. 1(e)). With a combination of six MUP, that is, flavour, emulsif*, colour, fibre, dextrose and firming, almost 90 % of the UPF products were identified (875 UPF products, 88·4 %, Fig. 1(f)).

## Discussion

### Principal findings

In the present study, MUP are assessed for the first time in a large sample of commercial products to elucidate the most successful combinations for UPF detection. The results enable consumers to strike the optimal individual balance between the number of MUP to remember on the one hand and the proportion of UPF items identified correctly on the other hand. Thus, already 58·4 % and 72·7 % of UPF can be detected with a single MUP, that is, flavour, and the MUP combination of flavour and emulsif*, respectively. Almost 90 % of UPF can be identified with the combination of six MUP, that is, flavour, emulsif*, colour, fibre, dextrose and firming. Future studies should assess whether consumers can successfully identify UPF in the supermarket by using this MUP-based approach. In particular, experiments should elucidate which number of MUP can be recalled and correctly applied to specific products.

### Comparison with other studies

In the current study, 53·9 % of the 1836 food products with ingredient lists are positive for at least one MUP and, therefore, defined as UPF. Using a similar approach in a large-scale database of foods and beverages available on the French market, 53·8 % of products with an ingredient list, that is, 68 110 out of 126 556, contained at least one food additive^(22)^. Interestingly, 17·8 % had one, 11·6 % two, 7·8 % three, 5·3 % four and 11·3 % five or more food additives^(22)^. In a study from Australia, 4794 out of 7322 food items (65·5 %) are defined as UPF^(23)^. Similarly, 67 % of 24 932 packaged food items from France are UPF^(14)^. In a study by the same authors, the percentage of MUP dextrose is similar to the current findings, whereas other MUP including protein isolates are more frequently observed on the French market as compared to the present UK sample^(24)^. In agreement with the current results, most MUP are present in less than 10 % of UPF items^(24)^. However, to the best of our knowledge, the present study is the first to assess which MUP combinations detect the maximum number of UPF products.

Added flavours are by far the most prevalent MUP being detected in 58·4 % of UPF. In accordance with the present findings, extracts/natural flavours and synthetic flavours are present in 42·7 % and 26·5 % of UPF, respectively, in a representative and weighted food offer found in overall French supermarkets^(24)^. Evidence has recently been presented that added flavours might induce overeating and body weight gain^(25)^. Thus, added flavours override homoeostatic control of food intake by the promotion of hedonic eating^(25)^. Furthermore, they impair the ability to predict nutrients in food items via disruption of flavour-nutrient learning^(25)^. Taking these results and our current findings into consideration, added flavours might not only be the MUP detecting the maximum number of UPF, but they might also be ingredients actively promoting obesity and its consequences.

Besides added flavours, emulsifiers, colouring agents, dextrose, whey and gluten are found in more than 10 % of all UPF. It has been suggested that some common food emulsifiers induce metabolic and chronic inflammatory disease by altering the gut microbiome and intestinal barrier^(26)^. Dextrose is a monosaccharide which is regarded as a free sugar when added to food items by the manufacturer^(27)^. Free sugars have been convincingly linked to body weight gain and, therefore, the WHO suggests to limit free sugar consumption to less than 10 % of total energy intake per day^(27)^. Moreover, the addition of gluten to a normal chow and a high-fat diet increases body weight and fat deposits without changing food intake in mice^(28)^. Whereas colour variety can enhance selection, no increase on continuous food intake has been demonstrated^(29)^. Furthermore, whey protein supplementation might improve body weight and total fat mass in overweight and obese subjects^(30)^. Together these data suggest that emulsifiers, dextrose and gluten are not only relevant as MUP, but they might also directly contribute to conditions favouring metabolic disease, that is, low-grade inflammation (emulsifiers), increased food intake (dextrose) and body weight gain (dextrose and gluten). Further studies need to elucidate their role, as well as the role of other MUP, in the development of metabolic disease in more detail.

### Health and policy implications

Besides overweight and obesity^(2)^, increased UPF consumption has been convincingly linked with other adverse outcomes. Thus, a significant 1·6-fold increase in all-cause mortality is detected in a Spanish prospective cohort study (*n* 19 899) if subjects with the highest UPF consumption are compared to the lowest UPF intake^(31)^. In agreement with these findings, all-cause and CVD mortality are increased 1·4-fold and 1·7-fold, respectively, in subjects from Italy presenting with a history of CVD (*n* 1171) when comparing the highest with the lowest quartile of UPF intake^(32)^. Risk for CVD is also significantly increased 1·2-fold in a large prospective cohort study from France (*n* 105 159) in the highest as compared to the lowest consumption category for UPF^(33)^. Dementia risk is increased 1·3-fold if the proportion of UPF in the diet increases by 10 % in subjects from the UK (*n* 72 083)^(34)^. Incident depression risk is increased 1·2-fold for a 10 % increase in UPF consumption in a study from France (*n* 26 730)^(35)^. Furthermore, the risk of inflammatory bowel disease increases 1·8-fold in a prospective cohort study in twenty-one low-, middle- and high-income countries (*n* 116 087) in the highest as compared to the lowest UPF consumption group^(36)^. Moreover, UPF consumption in the highest as compared to the lowest quartile is associated with a 1·2-fold and 1·1-fold increase in overall cancer mortality and morbidity, respectively, in a prospective UK study (*n* 197 426)^(37)^.

Therefore, consumers should recognise and consciously avoid UPF. The present study enables consumers to identify almost 80 % and 90 % of all UPF items by searching the ingredient lists for three and six MUP, respectively. This MUP-based approach considerably simplifies UPF detection. However, consumers still need to study ingredient lists to identify UPF and ingredient lists are not always available, for example, in food prepared outside the home. Therefore, public policy strategies should be implemented which enable consumers to avoid UPF items more easily without the need for studying ingredient lists. The WHO recommends the use of front-of-package labelling (FOPL) and reformulation of food products to create healthy food and drink environments^(38)^. FOPL like the Nutri-Score improves the ability of participants to rank products according to healthiness correctly and offers the potential to increase sales of healthy food items^(39,40)^. Furthermore, FOPL provides incentives for manufacturers to decrease the level of processing by changing product compositions^(41,42)^. The NOVA group of food items can be depicted by a FOPL^(43)^. Furthermore, a novel nutrition classification scheme combining level of processing and nutrient thresholds for Na and free sugars has recently been proposed^(23)^. Similarly, the Pan American Health Organization Nutrient Profile Model not only defines UPF but also sets thresholds for critical nutrients, including free sugars, Na, total fat, saturated fat, trans fat and sweeteners^(44)^. Identification of UPF items enables policymakers to reduce UPF consumption on a population level, for example, by taxation^(45,46)^. Real-world evaluation studies suggest that taxation of sugar-sweetened beverages reduces purchases and consumption of this important UPF category^(47)^. Further, public health policies to limit UPF intake besides FOPL and taxation include restrictions on the marketing of unhealthy food targeting children, regulation of school food environments and reexamination of agricultural subsidies^(44)^.

### Strengths and limitations of this study

Strengths of the current study include a large sample size of commercial food items, structured detection of UPF with defined MUP and analyses not only of single MUP but also of their combinations.

A limitation is that only the UK market was analysed and the distribution of MUP in UPF might be different in other countries. Furthermore, food items without an ingredient list had to be excluded from the analysis. In most cases, it can be assumed that these products are not UPF, for example, fruits, vegetables, and eggs, and, thus, their exclusion has no impact on the results of the current study. However, other food items without ingredients lists might be in part UPF, especially some types of alcoholic beverages.

It has been convincingly shown recently that detection of UPF items differs depending on the approach used and the selection of individual MUP^(48)^. Furthermore, food can also be UPF in the absence of MUP if processes like extrusion, hydrolysation and, pre-frying are applied^(3,13,14)^. Therefore, the MUP-based approach used in the current study might underestimate the proportion of UPF items. However, information on processes like extrusion, hydrolysation and pre-frying cannot be easily obtained by individual consumers in contrast to information about MUP which can be extracted from ingredient lists. Moreover, the possibility of hidden additives in food products, for example, compound ingredients not thoroughly described in the ingredient list, cannot be excluded and might underestimate the proportion of UPF items. On the other hand, some additives defined as MUP in the current study are not MUP in all circumstances, for example, maltodextrin if used as a stabiliser, which might incorrectly identify some food items as UPF. Moreover, the proportion of UPF detected by MUP and the number of MUP per product might depend on the type of food or food category. However, food groups are not studied separately in the present analysis, since its aim is to elucidate which MUP and their combinations are best suited to detect UPF over the whole range of food.

In the present study, food specifically targeting children has been excluded from the analysis, since the Oxford WebQ has only been developed for and used in adult participants. It is important to note in this context that in a recent study from France, 88 % of food specifically targeting children over the age of 3 years is UPF^(49)^. Furthermore, in a report from Portugal, 56 % of food products for children aged 0 to 3 years are UPF^(50)^.

### Conclusions

Based on research of commercial food items from the two market leaders of groceries in the UK, the present study enables consumers to identify almost 80 % and 90 % of all UPF items by searching the ingredient lists for three and six MUP, respectively. These findings might help consumers to make healthier choices when shopping for groceries by avoiding UPF which has been consistently linked with a broad range of adverse outcomes. In addition, they can also help researchers in food classification in dietary surveys.

## Supporting information

Neumann et al. supplementary material Neumann et al. supplementary material

## Supplementary material

For supplementary material accompanying this paper visit https://doi.org/10.1017/S1368980023002185

## References

[1] Seidell JC & Halberstadt J (2015) The global burden of obesity and the challenges of prevention. Ann Nutr Metab 66, Suppl. 2, 7–12. 10.1159/000375143 26045323

[2] Lane MM , Davis JA , Beattie S et al. (2021) Ultraprocessed food and chronic noncommunicable diseases: a systematic review and meta-analysis of 43 observational studies. Obes Rev 22, e13146. 33167080 10.1111/obr.13146

[3] Monteiro CA , Cannon G , Moubarac J-C et al. (2017) The UN decade of nutrition, the NOVA food classification and the trouble with ultra-processing. Public Health Nutr 21, 5–17. 28322183 10.1017/S1368980017000234 PMC10261019

[4] Baker P , Machado P , Santos T et al. (2020) Ultra-processed foods and the nutrition transition: global, regional and national trends, food systems transformations and political economy drivers. Obes Rev 21, e13126. 32761763 10.1111/obr.13126

[5] Ministry of Health of Brazil, Secretariat of Health Care & Primary Health Care Department (2015) Dietary Guidelines for the Brazilian Population. https://bvsms.saude.gov.br/bvs/publicacoes/dietary_guidelines_brazilian_population.pdf (accessed June 2023).

[6] Health Canada (2019) Canada’s Dietary Guidelines for Health Professionals and Policy Makers. https://food-guide.canada.ca/sites/default/files/artifact-pdf/CDG-EN-2018.pdf (accessed June 2023).

[7] Ministerio de Salud Pública del Ecuador y FAO (2021) Documento Técnico de las Guías Alimentarias Basadas en Alimentos (GABA) del Ecuador (Technical Document of the Food-Based Dietary Guidelines (FBDG) of Ecuador). https://www.fao.org/3/ca9955es/ca9955es.pdf (accessed June 2023).

[8] Santé Publique France (2019) Recommendations Concerning Diet, Physical Activity and Sedentary Behaviour for Adults. https://www.santepubliquefrance.fr/determinants-de-sante/nutrition-et-activite-physique/documents/rapport-synthese/recommandations-relatives-a-l-alimentation-a-l-activite-physique-et-a-la-sedentarite-pour-les-adultes (accessed June 2023).

[9] The Israel Ministry of Health (2019) Nutritional Recommendation of the Isreal Ministry of Health. https://www.health.gov.il/PublicationsFiles/dietary%20guidelines%20EN.pdf (accessed June 2023).

[10] Food and Agriculture Organization of the United Nations (2022) Food-Based Dietary Guidelines – Japan. https://www.fao.org/nutrition/education/food-dietary-guidelines/regions/countries/japan/en/ (accessed June 2023).

[11] McIntyre L , Jackson A , Carr H et al. (2020) Eating and Activity Guidelines for New Zealand Adults. https://www.health.govt.nz/system/files/documents/publications/eating-activity-guidelines-new-zealand-adults-updated-2020-oct22.pdf (accessed June 2023).

[12] Lázaro Serrano ML & Domínguez Curi CA (2020) Guías Alimentarias para la Población Peruana (Dietary Guidelines for the Peruvian Population). https://repositorio.ins.gob.pe///handle/20.500.14196/1247 (accessed June 2023).

[13] Monteiro CA , Cannon G , Levy RB et al. (2019) Ultra-processed foods: what they are and how to identify them. Public Health Nutr 22, 936–941. 30744710 10.1017/S1368980018003762 PMC10260459

[14] Davidou S , Christodoulou A , Fardet A et al. (2020) The holistico-reductionist Siga classification according to the degree of food processing: an evaluation of ultra-processed foods in French supermarkets. Food Funct 11, 2026–2039. 32083627 10.1039/c9fo02271f

[15] Liu B , Young H , Crowe FL et al. (2011) Development and evaluation of the Oxford WebQ, a low-cost, web-based method for assessment of previous 24 h dietary intakes in large-scale prospective studies. Public Health Nutr 14, 1998–2005. 21729481 10.1017/S1368980011000942

[16] Perez-Cornago A , Pollard Z , Young H et al. (2021) Description of the updated nutrition calculation of the Oxford WebQ questionnaire and comparison with the previous version among 207,144 participants in UK Biobank. Eur J Nutr 60, 4019–4030. 33956230 10.1007/s00394-021-02558-4 PMC8437868

[17] Kantar (2022) Grocery Market Share. https://www.kantar.com/campaigns/grocery-market-share (accessed December 2022).

[18] Greenwood DC , Hardie LJ , Frost GS et al. (2019) Validation of the Oxford WebQ online 24-h dietary questionnaire using biomarkers. Am J Epidemiol 188, 1858–1867. 31318012 10.1093/aje/kwz165 PMC7254925

[19] Timon CM , van den Barg R , Blain RJ et al. (2016) A review of the design and validation of web- and computer-based 24-h dietary recall tools. Nutr Res Rev 29, 268–280. 27955721 10.1017/S0954422416000172

[20] Conrad J , Koch SAJ & Nöthlings U (2018) New approaches in assessing food intake in epidemiology. Curr Opin Clin Nutr Metab Care 21, 343–351. 29939967 10.1097/MCO.0000000000000497

[21] European Parliament Council of the European Union (2011) Regulation (EU) No 1169/2011 of the European Parliament and of the Council of 25 October 2011 on the Provision of Food Information to Consumers, Amending Regulations (EC) No 1924/2006 and (EC) No 1925/2006 of the European Parliament and of the Council, and Repealing Commission Directive 87/250/EEC, Council Directive 90/496/EEC, Commission Directive 1999/10/EC, Directive 2000/13/EC of the European Parliament and of the Council, Commission Directives 2002/67/EC and 2008/5/EC and Commission Regulation (EC) No 608/2004: L 304/18. https://eur-lex.europa.eu/LexUriServ/LexUriServ.do?uri=OJ:L:2011:304:0018:0063:en:PDF (accessed October 2023).

[22] Chazelas E , Deschasaux M , Srour B et al. (2020) Food additives: distribution and co-occurrence in 126,000 food products of the French market. Sci Rep 10, 3980. 32132606 10.1038/s41598-020-60948-w PMC7055242

[23] Dickie S , Woods J , Machado P et al. (2023) A novel food processing-based nutrition classification scheme for guiding policy actions applied to the Australian food supply. Front Nutr 10, 1071356. 36742430 10.3389/fnut.2023.1071356 PMC9895835

[24] Davidou S , Christodoulou A , Frank K et al. (2021) A study of ultra-processing marker profiles in 22,028 packaged ultra-processed foods using the Siga classification. J Food Compos 99, 103848.

[25] Neumann NJ & Fasshauer M (2022) Added flavors: potential contributors to body weight gain and obesity? BMC Med 20, 417. 36319974 10.1186/s12916-022-02619-3 PMC9623908

[26] Siena MD , Raoul P , Costantini L et al. (2022) Food emulsifiers and metabolic syndrome: the role of the gut microbiota. Foods 11, 15. 10.3390/foods11152205 PMC9331555 35892789

[27] World Health Organization (2015) Guideline: Sugars Intake for Adults and Children. https://www.ncbi.nlm.nih.gov/books/NBK285537/ (accessed June 2023). 25905159

[28] Freire RH , Fernandes LR , Silva RB et al. (2016) Wheat gluten intake increases weight gain and adiposity associated with reduced thermogenesis and energy expenditure in an animal model of obesity. IJO 40, 479–486. 10.1038/ijo.2015.204 26443339

[29] Piqueras-Fiszman B & Spence C (2014) Colour, pleasantness, and consumption behaviour within a meal. Appetite 75, 165–172. 24462488 10.1016/j.appet.2014.01.004

[30] Wirunsawanya K , Upala S , Jaruvongvanich V et al. (2018) Whey protein supplementation improves body composition and cardiovascular risk factors in overweight and obese patients: a systematic review and meta-analysis. J Am Coll Nutr 37, 60–70. 29087242 10.1080/07315724.2017.1344591

[31] Rico-Campà A , Martínez-González MA , Alvarez-Alvarez I et al. (2019) Association between consumption of ultra-processed foods and all cause mortality: SUN prospective cohort study. BMJ 365, l1949. 31142450 10.1136/bmj.l1949 PMC6538973

[32] Bonaccio M , Costanzo S , Di Castelnuovo A et al. (2022) Ultra-processed food intake and all-cause and cause-specific mortality in individuals with cardiovascular disease: the Moli-Sani study. Eur Heart J 43, 213–224. 34849691 10.1093/eurheartj/ehab783

[33] Srour B , Fezeu LK , Kesse-Guyot E et al. (2019) Ultra-processed food intake and risk of cardiovascular disease: prospective cohort study (NutriNet-Santé). BMJ 365, l1451. 31142457 10.1136/bmj.l1451 PMC6538975

[34] Li H , Li S , Yang H et al. (2022) Association of ultraprocessed food consumption with risk of dementia: a prospective cohort. Neurology 99, e1056–e1066. 36219796 10.1212/WNL.0000000000200871

[35] Adjibade M , Julia C , Allès B et al. (2019) Prospective association between ultra-processed food consumption and incident depressive symptoms in the French NutriNet-Santé cohort. BMC Med 17, 78. 30982472 10.1186/s12916-019-1312-y PMC6463641

[36] Narula N , Wong ECL , Dehghan M et al. (2021) Association of ultra-processed food intake with risk of inflammatory bowel disease: prospective cohort study. BMJ 374, n1554. 34261638 10.1136/bmj.n1554 PMC8279036

[37] Chang K , Gunter MJ , Rauber F et al. (2023) Ultra-processed food consumption, cancer risk and cancer mortality: a large-scale prospective analysis within the UK Biobank. EClinicalMedicine 56, 101840. 36880051 10.1016/j.eclinm.2023.101840 PMC9985039

[38] World Health Organization & Regional Office for Europe (2015) European Food and Nutrition Action Plan 2015–2020. https://apps.who.int/iris/bitstream/handle/10665/329405/9789289051231-eng.pdf?sequence=1&isAllowed=y (accessed June 2023).

[39] De Temmerman J , Heeremans E , Slabbinck H et al. (2021) The impact of the Nutri-Score nutrition label on perceived healthiness and purchase intentions. Appetite 157, 104995. 33068665 10.1016/j.appet.2020.104995

[40] Packer J , Russell SJ , Ridout D et al. (2021) Assessing the effectiveness of front of pack labels: findings from an online randomised-controlled experiment in a representative British Sample. Nutrients 13, 3. 10.3390/nu13030900 PMC7999818 33802115

[41] Van der Bend DLM , Jansen L , van der Velde G et al. (2020) The influence of a front-of-pack nutrition label on product reformulation: a 10-year evaluation of the Dutch choices programme. Food Chem 6, 100086. 10.1016/j.fochx.2020.100086 PMC7152661 32300755

[42] Ter Borg S , Steenbergen E , Milder IEJ et al. (2021) Evaluation of Nutri-Score in relation to dietary guidelines and food reformulation in the Netherlands. Nutrients 13, 12. 10.3390/nu13124536 PMC8704310 34960088

[43] Valenzuela A , Zambrano L , Velásquez R et al. (2022) Discrepancy between food classification systems: evaluation of Nutri-Score, NOVA classification and Chilean front-of-package food warning labels. IJERPH 19, 22. 10.3390/ijerph192214631 PMC9690311 36429354

[44] Pan American Health Organization & World Health Organization (2016) Nutrient Profile Model. https://iris.paho.org/bitstream/handle/10665.2/18621/9789275118733_eng.pdf?sequence=9&isAllowed=y (accessed June 2023).

[45] Passos CMD , Maia EG , Levy RB et al. (2020) Association between the price of ultra-processed foods and obesity in Brazil. NMCD 30, 589–598. 32139251 10.1016/j.numecd.2019.12.011

[46] Langellier BA , Stankov I , Hammond RA et al. (2022) Potential impacts of policies to reduce purchasing of ultra-processed foods in Mexico at different stages of the social transition: an agent-based modelling approach. Public Health Nutr 25, 1711–1719. 34895382 10.1017/S1368980021004833 PMC7612742

[47] Teng AM , Jones AC , Mizdrak A et al. (2019) Impact of sugar-sweetened beverage taxes on purchases and dietary intake: systematic review and meta-analysis. Obes Rev 20, 1187–1204. 31218808 10.1111/obr.12868 PMC9285619

[48] Zancheta Ricardo C , Duran AC , Grilo MF et al. (2022) Impact of the use of food ingredients and additives on the estimation of ultra-processed foods and beverages. Front Nutr 9, 1046463. 36704802 10.3389/fnut.2022.1046463 PMC9872514

[49] Richonnet C , Mosser F , Favre E et al. (2021) Nutritional quality and degree of processing of children’s foods assessment on the French market. Nutrients 14, 171. 35011047 10.3390/nu14010171 PMC8747148

[50] De Araújo CRB , da S Ribeiro KD , de Oliveira AF et al. (2021) Degree of processing and nutritional value of children’s food products. Public Health Nutr 24, 5977–5984. 34494515 10.1017/S1368980021003876 PMC11096954

