# Supplementary material for: Flavour, emulsifiers and colour are the most frequent markers to detect food ultra-processing in a UK food market analysis
Source: Public Health Nutr. 2023 Oct 19;26(12):3303–10. doi: 10.1017/S1368980023002185 (PMC10755427; doi:10.1017/S1368980023002185)
Supplement: Neumann et al. supplementary material [file S1368980023002185sup001.docx]

**Supplementary Table 1**

MUPs and their individual compounds within the nine categories^1^

| **Category** | **MUPs** | **Individual compound** |
| --- | --- | --- |
| **Cosmetic additives** | | |
| 1. Flavours | 1. Flavour | Flavor  Flavour |
| 1. Flavour enhancers | 1. E62* | E620 – E629 |
|  | 1. E63* | E630 – E637 |
|  | 1. E640 | E640 |
|  | 1. E650 | E650 |
|  | 1. Flavour enhancer | Flavour enhancer |
|  | 1. Glutam* | [Calcium diglutamate](https://en.wikipedia.org/wiki/Calcium_diglutamate)  Glutamic acid  [Magnesium diglutamate](https://en.wikipedia.org/wiki/Magnesium_diglutamate)  [Monoammonium glutamate](https://en.wikipedia.org/wiki/Monoammonium_glutamate)  [Monopotassium glutamate](https://en.wikipedia.org/wiki/Monopotassium_glutamate)  [Monosodium glutamate](https://en.wikipedia.org/wiki/Monosodium_glutamate) |
|  | 1. Glycine | Glycine  [Glycine](https://en.wikipedia.org/wiki/Glycine) and its sodium salt |
|  | 1. Guanyl* | [Calcium guanylate](https://en.wikipedia.org/wiki/Calcium_guanylate)  [Dipotassium guanylate](https://en.wikipedia.org/wiki/Dipotassium_guanylate)  [Disodium guanylate](https://en.wikipedia.org/wiki/Disodium_guanylate)  [Guanylic acid](https://en.wikipedia.org/wiki/Guanylic_acid)  Sodium guanylate |
|  | 1. Inosin* | [Calcium inosinate](https://en.wikipedia.org/wiki/Calcium_inosinate)  [Dipotassium inosinate](https://en.wikipedia.org/wiki/Dipotassium_inosinate)  [Disodium inosinate](https://en.wikipedia.org/wiki/Disodium_inosinate)  [Inosinic acid](https://en.wikipedia.org/wiki/Inosinic_acid) |
|  | 1. Maltol | Ethyl maltol  Maltol |
|  | 1. MSG | MSG |
|  | 1. Ribonucleotide* | [Calcium 5'-ribonucleotides](https://en.wikipedia.org/wiki/Calcium_5%27-ribonucleotides)  [Disodium 5'-ribonucleotides](https://en.wikipedia.org/wiki/Disodium_5%27-ribonucleotide) |
|  | 1. Zinc acetate | Zinc acetate |
| 1. Colouring agents | 1. Colour | Colour  Colour stabiliser |
|  | 1. Dye | Dye |
| 1. Sweeteners | 1. Acesulfame | Acesulfame K  Salt of aspartame-acesulfame |
|  | 1. Advantame | Advantame |
|  | 1. Aspartame | Aspartame  Salt of aspartame-acesulfame |
|  | 1. Cyclam* | Cyclamate  Cyclamic acid |
|  | 1. E420 | E420 |
|  | 1. E421 | E421 |
|  | 1. E95* | E950 – E959 |
|  | 1. E96* | E960 – E969 |
|  | 1. Erythritol | Erythritol |
|  | 1. Isomalt | Isomalt |
|  | 1. Lactitol | Lactitol |
|  | 1. Maltitol | Maltitol  Maltitol syrup |
|  | 1. Mannitol | Mannitol |
|  | 1. Neohesperidine | Neohesperidine DC |
|  | 1. Neotame | Neotame |
|  | 1. Polyglycitol | Polyglycitol |
|  | 1. Saccharin | Saccharin |
|  | 1. Sorbitol | Sorbitol  Sorbitol syrup |
|  | 1. Steviol | Steviol glycoside |
|  | 1. Sucralose | Sucralose |
|  | 1. Sweetener | Sweetener |
|  | 1. Thaumatin | Thaumatin |
|  | 1. Xylitol | Xylitol |
| 1. Processing aids | 1. Bulking | Anti-bulking  Bulking agent |
|  | 1. Caking | Anti-caking agent  Anticaking agent |
|  | 1. Carbonating | Carbonating agent |
|  | 1. Emulsif* | Emulsifier  Emulsifying salts |
|  | 1. Firming | Firming agent |
|  | 1. Foaming | Anti-foaming agent  De-foaming agent  Foaming agent |
|  | 1. Gelling | Gelling agent |
|  | 1. Glazing | Glazing agent |
|  | 1. Humectant | Humectant |
|  | 1. Sequestrant | Sequestrant |
|  | 1. Thickener | Thickener |
| **Non-culinary ingredients** | | |
| 1. Varieties of sugar | 1. Barley malt extract | Barley malt extract^#^ |
|  | 1. Dextrose | Dextrose  Polydextrose^#^ |
|  | 1. Fructose | Fructose  Fructose-glucose syrup^#^ Glucose-fructose syrup^#^  High-fructose corn syrup |
|  | 1. Invert* | Inverted refiners syrup^#^  Inverted sugar syrup^#^  Invert sugar |
|  | 1. Lactose | Lactose |
|  | 1. Maltodextrin | Maltodextrin |
| 1. Modified oils | 1. Hydrogenated | Hydrogenated oil |
|  | 1. Interesterified | Interesterified oil |
| 1. Protein sources | 1. Casein | Casein |
|  | 1. Gluten | Gluten |
|  | 1. Hydrolysed | Hydrolysed protein |
|  | 1. Isolate* | Isolated soy protein  Protein isolate^#^  Soy protein isolate |
|  | 1. Mechanically separated meat | Mechanically separated meat |
|  | 1. Whey | Dried whey^#^  Whey^#^  Whey derivatives^#^  Whey permeate^#^  Whey powder^#^  Whey protein  Whey solids^#^ |
| 1. Fibres | 1. Fibre | Fibre  Fibre isolate^#^  Insoluble fibre  Soluble fibre |

^1^Individual compounds are based on NOVA group 4^(3,13)^. *Indicates that all variations of the word are possible (e.g., glutamic and glutamate are possible for glutam*). ^#^Indicates individual compounds which are not literally mentioned in the two publications^(3,13)^ but which are related to the ultra-processing compounds mentioned.
